# Characterization of Clinical *Salmonella entericas* Trains in Huzhou, China

**DOI:** 10.1155/2022/7280376

**Published:** 2022-06-21

**Authors:** Deshun Xu, Lei Ji, Wei Yan, Liping Chen

**Affiliations:** Huzhou Center for Disease Control and Prevention, 999 Changxing Road, Huzhou, Zhejiang 313000, China

## Abstract

**Background:**

*Salmonella enterica* subspecies *enterica* causes salmonellosis in humans and animals and is an important antecedent of food infections worldwide. This study collected 105 clinical *S*. *enterica* isolates from diarrhoea samples from six sentinel hospitals for active surveillance of foodborne diseases in Huzhou, China, between 2018 and 2020. These represented all the *Salmonella* isolates collected in Huzhou during that period.

**Methods:**

The isolates were characterized by serovar determination, antimicrobial susceptibility tests, and pulse-field gel electrophoresis (PFGE) typing.

**Results:**

The 105 *Salmonella* strains were mainly *S*. *typhimurium* (35.24%, 95% CI from 25.95 to 44.53%) and *S*. *enteritidis* (18.10%, 95% CI from 10.61 to 25.58%). Testing indicated that the resistance rate of the *Salmonella* strains ranged from 0.00% to 70.48%, and the highest resistance rate was for ampicillin (70.48%; 74/105), followed by tetracycline (67.62%; 71/105) and doxycycline (65.71%; 69/105). Following *Xba*I digestion, the 105 strains yielded 93 PFGE patterns, and 15 clones had similarity values >85.00%.

**Conclusions:**

Our analyses revealed the serovar distribution of isolates recovered from diarrhoea patients and the characteristics of resistant strains in Huzhou from 2018 to 2020. Our results highlight a serovar shift and a concerning number of multidrug-resistant (MDR) strains. Continued surveillance of *Salmonella* and their MDR profiles and efforts to control the rapid increase in antimicrobial resistance among *Salmonella* in Huzhou are needed.

## 1. Introduction

*Salmonella* is an important zoonotic pathogen in Enterobacteriaceae. It can survive for long periods in meat, eggs, and related products, and frequently causes human gastroenteritis and other types of food poisoning, especially in developing countries [1]. *Salmonella* can contaminate the entire food chain and eventually infect people during *Salmonella* outbreaks [2]. *Salmonella* is the major pathogen causing foodborne diseases [3, 4]. Salmonellosis causes approximately 93.8 million cases of gastroenteritis and 155,000 deaths per year worldwide [5] and often acts in coinfection with other enteric pathogens [6]. *Salmonella* infection-related hospitalizations and deaths dominated foodborne disease outbreaks in the United States in 2011, in both active and passive surveillance systems [3]. In China, 9.035 million cases of foodborne nontyphoid salmonellosis were reported every year, with 792 deaths every year [7]. Salmonellosis affects both human health and the economy.

Serotyping is the traditional method for subtyping and differentiating *Salmonella* isolates based on the Kauffmann–White (KW) scheme. Over 2,700 *Salmonella* serotypes are known [8]. However, only 40∼50 serotypes have been isolated from humans, animals, and food [9]. The main serotypes of gastroenteritis cases are *Salmonella enterica serotypes enteritidis* and *typhimurium*, while the main serotype in animals/animal products is Indiana [10, 11]. While the traditional serotyping method is mainly used to identify *Salmonella* serotypes, it cannot identify different strains of the same serotype [12]. Pulsed-field gel electrophoresis (PFGE) determines the kinship of strains isolated by other means, based on the principle that individuals from the same parent have common genetic material and the same PFGE fingerprints. Determining the etiological relationships among cases can compensate for serotyping deficiencies [12, 13].

Antibiotics are commonly used to treat *Salmonella* infection, but extensive use of antibiotics has increased the number of *Salmonella* serotypes resistant to various antibiotics [2, 14]. Outbreaks of drug-resistant *Salmonella* (and changes in the drug resistance spectrum) are difficult to treat and threaten public health. To understand *Salmonella'*s serotypes, drug resistance, and molecular typing characteristics in Huzhou, 105 strains of *Salmonella* isolated from diarrhoea cases in Huzhou from 2018 to 2020 were typed serologically, and drug resistance analysis and PFGE typing of the strains were performed.

## 2. Materials and Methods

### 2.1. Bacterial Isolates

The study examined 105 *Salmonella* strains isolated from six active foodborne surveillance sentinel hospitals in Huzhou, Zhejiang from 2018 to 2020 (31, 49, and 25 strains, respectively). The standard *Salmonella enterica* strain for PFGE is serotype Braenderup (H9812), from the Zhejiang Center for Disease Control and Prevention.

### 2.2. Isolation and Identification of Bacteria

Diarrhoea (anus swab) specimens were grown in selenite brilliant green sulfa enrichment broth and then inoculated in *Salmonella* chromogenic medium for separation. Suspicious colonies were identified after the pure culture. Finally, a Vitekautomatic bacterial identification instrument (bio Mérieux, Inc., Marcy-l'Étoile, France) was used for the biochemical identification of the *Salmonella* strains.

### 2.3. Serotyping

The isolated, purified positive strains were inoculated on blood plates and cultured at 37 for 18 h. A single colony was selected for O antigen slide agglutination. Then, *Salmonella* H phase induced agar was used for H1 and H2 phase flagellar induction and serum agglutination. The K-W serotyping table was searched for the obtained antigen formula to determine the serotype. Normal saline was used as a self-coagulation control.

### 2.4. Antimicrobial Susceptibility Testing

The antimicrobial susceptibilities of the 105 clinical *Salmonella* strains were tested using the broth microdilution method and classified as sensitive, intermediate, or resistant according to the Clinical and Laboratory Standards Institute (CLSI) breakpoints for *Salmonella* strains. *Escherichia coli* ATCC 25922 was used as a control. The results were analysed according to the CLSI breakpoints.

The identification board contains the following 30 antibiotics: ampicillin (AMP), AMP/sulbactam (AMS), tetracycline (TET), chloramphenicol (CHL), cotrimoxazole (SXT), cefazolin (CFZ), cefotaxime (CTX), ceftazidime (CAZ), cefoxitin (CFX), gentamicin (GEN), imipenem (IMI), naphthalic acid (NAL), azithromycin (AZI), sulfisoxazole (SuL), ciprofloxacin (CIP), amoxicillin-clavulanic acid (AMC), cefotaxime-clavulanic acid (CTX/C), ceftazidime-clavulanic acid (CAZ/C), polymyxin E (CT), polymyxin B (PB), minocycline (MIN), amikacin (AN), aztreonam (ATM), cefepime (FEP), meropenem (MEM), levofloxacin (LEV), doxycycline (DOX), kanamycin (KAN), streptomycin (STR), and gemifloxacin (GMI).

### 2.5. Pulsed-Field Gel Electrophoresis

The *Salmonella* isolates were subjected to PFGE analysis according to the standard nontyphoid *Salmonella* PFGE method of the National Pathogen Identification Network. The *Salmonella* standard strain H9812 was used as the standard. Briefly, the chromosomal DNA was digested with *Xba*I. The restriction fragments were resolved with 1% SeaKem gold agarose gels in 0.5% Tris-boric acid-EDTA buffer using the CHEF Mapper XA system (Bio-Rad Laboratories, Richmond, CA, USA). The PFGE patterns were analyzed using BIONUMERICS 7.1. Clustering was performed using the unweighted pair group method and the Dice correlation coefficient with a position tolerance of 1.5%. Clusters were defined using a 90% similarity cutoff [15].

## 3. Results

### 3.1. Serotyping

From 2018 to 2020, the Huzhou Foodborne Disease Surveillance System isolated 105 *Salmonella* strains. These were divided into 26 serotypes belonging to six groups: groups B (6 serotypes), C1 (7 serotypes), C2 (1 serotype), C3 (4 serotypes), D (4 serotypes), and E1 (4 serotypes). There were 46, 26, and 15 strains from groups B, D, and E1, respectively. *S*. *enterica* serovar *typhimurium* was the most common in group B. The prevalence was 35.24% (95% CI, from 25.95 to 44.53%). The prevalence of *S*. *enterica* serovar *enteritidis*, the most common in group D, was 18.10% (95% CI, 10.61–25.58). *S*. *enterica* serovar London, however, was the most common in group E1 with a prevalence of 8.57% (95%CI, 3.13–14.01). *Salmonella* Thompson and *Salmonella* Tennessee, however, were the most common in group C with a prevalence of 2.86% (95% CI, 0–6.1). The other *Salmonella* strains accounted for low proportions (Table 1).

### 3.2. Antimicrobial Resistance Profile

Table 2 shows the results of drug sensitivity testing of the 105 *Salmonella* strains to 30 antibiotics. The sensitivity ranged from 19.05% to 100%. The greatest sensitivity was to imipenem (100%), followed by azithromycin and amikacin (both 98.10%). The sensitivities to cefoxitin, cefotaxime-clavulanic acid, ceftazidime-clavulanic acid, aztreonam, cefepime, meropenem, and kanamycin all exceeded 90.00%. The rates for levofloxacin and AMP/sulbactam were 66.67% and 60.95%, respectively. The drug resistance rates for the 30 antibiotics ranged from 0.00% to 70.48%. AMP (70.48%) had the highest drug resistance rate, followed by TET (67.62%) and DOX (65.71%).

Of the 105 *Salmonella* strains, 80 were resistant to three or more antibiotics, and the total multiple drug resistance (MDR) rate was 76.19% (80/105). Drug resistance profiles were identified for 44 of the 105 *Salmonella* strains; the dominant drug resistance profile was AMP-TET-NAL DOX-STR (8 strains). The *Salmonella* resistant to five antibiotics accounted for 65.91% (29/44) of the MDR strains, and the most drug-resistant strains were two *Salmonella* strains detected in 2019; both were resistant to 12 antibiotics (Table 3).

### 3.3. PFGE and Cluster Analysis

**Figure 1:**
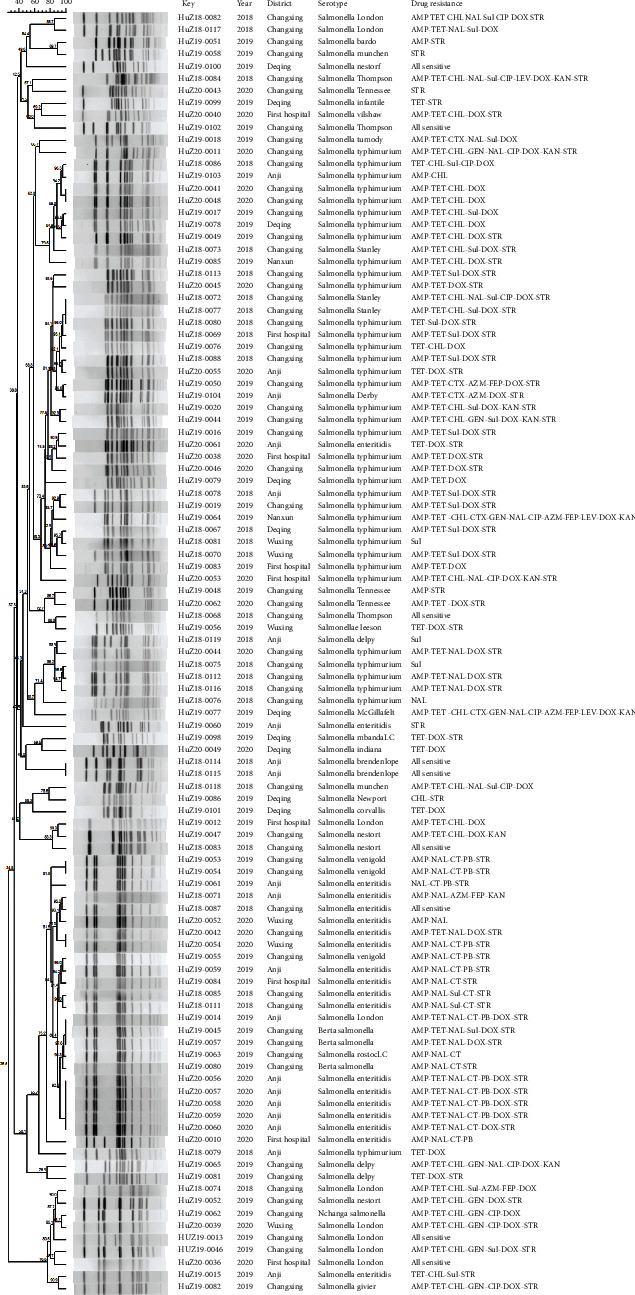
PFGE cluster analysis of 105 *Salmonella* strains collected from 2018 to 2020 in six sentinel hospitals from Huzhou, Zhejiang, China.

The 105 *Salmonella* strains were digested with the restriction endonuclease *Xba*I, and PFGE and cluster analysis were performed for all 105 strains (Figure 1). The band pattern similarity was 28.5% to 100.0%. Based on the number and location of bands, there were 93 different PFGE types, with one type containing up to five strains (*Salmonella enteritidis*, isolated in 2020). Fifteen clones had similarities exceeding 85.00%. Different PFGE bands may occur within the same serotype. Each serovar corresponded to a single clade, while a few isolates clustered in other serovar clades. *Salmonella enteritis* and *Salmonella typhimurium* showed clusters were observed.

## 4. Discussion

*Salmonella* is an important and widespread zoonotic pathogen that causes food poisoning and infectious diarrhoea [16]. About 70∼80% of patients with foodborne diseases in China have *Salmonella* infection, mainly nontyphoid *Salmonella* [17, 18]. All *Salmonella* serotypes can cause potentially life-threatening diseases. Therefore, knowledge of the distribution of *Salmonella* serotypes in a given area can help prevent *Salmonella* epidemics. The 105 *Salmonella* strains isolated by the Huzhou Food-borne Disease Surveillance System from 2018 to 2020 were all nontyphoid *Salmonella*. Twenty-six serotypes were isolated, among which *Salmonella typhimurium* was the most common, followed by *Salmonella enteritidis*; these are the dominant food-borne *Salmonella* serotypes in many parts of China [19, 20]. With the improvement in living standards, food consumption is becoming increasingly diversified. The food safety hazards caused by *Salmonella* are also increasing. Therefore, continuous *Salmonella* monitoring is necessary.

Bacterial drug resistance has become an important problem. The widespread use of antibiotics in agriculture and the irrational use of antibiotics in clinical practice lead to drug resistance in bacteria, including *Salmonella*. We showed that the drug resistance of *Salmonella* in the Huzhou area was serious; only 7 of the 105 *Salmonella* strains were sensitive to all 30 antibiotics and the remaining 98 strains were resistant to at least 1 antibiotic. Four antibiotics had drug resistance rates of over 50%: AMP (70.48%; 74/105), TET (67.62%; 71/105), DOX (65.71; 69/105), and STR (62.86; 66/105). They found similar to the drug resistance rates of *Salmonella* seen in other cities [12, 21, 22] The drug resistance rate to NAL in this study (36.19%) was different from that of Zhang et al. [22] (66.67%), which may be related to the difference in clinical medication in different regions. The drug resistance rate to cephalosporins was low, consistent with other reports [23].

The multidrug resistance of *Salmonella* is becoming increasingly serious. Of the 105 *Salmonella* strains, 80 were MDR strains. They were highly resistant to AMP and TET; 29 were resistant to six or more antibiotics, and one was resistant to 12 antibiotics. These MDR data are consistent with domestic reports [19]. Drug resistance monitoring of *Salmonella* helps elucidate temporal changes in drug resistance and can guide clinical use. Supervision of food production and processing should also be enhanced to prevent the spread of MDR strains.

PFGE analyzes the relationships among strains at the molecular level and can monitor, trace, and identify strains [24]. It is considered the “gold standard” for bacterial molecular typing because of its high repeatability and reliability. Using PFGE, a *Salmonella* database can be established to trace the source of foodborne disease outbreaks quickly, prevent the spread of disease and clarify the genetic relationships among *Salmonella* from different regions and years, and assess the epidemiological characteristics of *Salmonella*. This study shows that the patterns of *S*. *enteriti*s and *S*. *enterica typhimurium* showed two clusters. The molecular types of *Salmonella typhimurium* were mainly clustered in the upper half of Figure 1, while *Salmonella enteritidis* was mainly clustered in the lower half, consistent with Zhang et al. [22].

Other *Salmonella* types, such as *Salmonella* Zvenigorod, were also clustered, although not in large numbers. These clusters exist across regions and years, posing challenges to the prevention of foodborne outbreaks.

While the molecular types of different strains of the same serotype are similar, they are not completely consistent, which may be due to the horizontal transfer of antigen-determining genes between strains with distant genetic relationships; although the serotype is the same, there are obvious genetic differences [25]. We also found that the antibiotic resistance of strains with similar molecular types was highly comparable, such as HUZ20-56-60 and other strains. In some cases, the drug resistance spectrum can be determined by the molecular type.

## 5. Conclusions

This study analyzed the characteristics of *Salmonella enteritis* strains in diarrhoea samples from patients in Huzhou, Zhejiang. Different serotypes were detected in the clinical isolates. Drug resistance in *Salmonella typhimurium* was serious in Huzhou and multidrug-resistant strains were common. It is necessary to pay close attention to the emergence of antimicrobial-resistant strains and enhance antimicrobial management. The data in this study will be useful for controlling and treating food-borne illnesses caused by *Salmonella enterica* in Huzhou, Zhejiang.

## Figures and Tables

**Table 1:** Number of isolates and prevalence at 95% CI of salmonella serotypes in 105 *Salmonella* strains collected from 2018 to 2020 in six sentinel hospitals from Huzhou, Zhejiang, China.

Group	Serotype	Isolates	Prevalence (%)	95% CI
B	*Salmonella* Stanley	3	2.86	0–6.1
	*Salmonella* Delpy	3	2.86	0–6.1
	*Salmonella typhimurium*	37	35.24	25.95–44.53
	*Salmonella* Indiana	1	0.95	0–2.84
	*Salmonella* Tumody	1	0.95	0–2.84
	*Salmonella* Derby	1	0.95	0–2.84

C1	*Salmonella* Brendenlope	2	1.91	0–4.56
	*Salmonellae* Leeson	1	0.95	0–2.84
	*Salmonella* Thompson	3	2.86	0–6.1
	*Salmonella* Vilshaw	1	0.95	0–2.84
	*Salmonella* Infantile	1	0.95	0–2.84
	*Salmonella* Mbandaka	1	0.95	0–2.84
	*Salmonella* Tennessee	3	2.86	0–6.1

C2	*Salmonella* Munchen	2	1.91	0–4.56

C3	*Salmonella* Bardo	1	0.95	0–2.84
	*Salmonella* McGillafelt	1	0.95	0–2.84
	*Salmonella* Newport	1	0.95	0–2.84
	*Salmonella* Corvallis	1	0.95	0–2.84

D	*Salmonella enteritidis*	19	18.10	10.61–25.58
	Berta *Salmonella*	3	2.86	0–6.1
	*Salmonella* venigold	3	2.86	0–6.1
	*Salmonella* rostock	1	0.95	0–2.84

E1	Nchanga salmonella	1	0.95	0–2.84
	*Salmonella* nestorf	4	3.81	0.09–7.53
	*Salmonella* London	9	8.57	3.13–14.01
	*Salmonella* givier	1	0.95	0–2.84
Total		105	100.00	

**Table 2:** Antimicrobial susceptibility of *Salmonella* strains (*n* = 105) collected from 2018 to 2020 in six sentinel hospitals from Huzhou, Zhejiang, China.

Antimicrobial	Sensitive (n, %)	Intermediate (n, %)	Resistant (n, %)
AMP	31 (29.52)	0 (0.00)	74 (70.48)
AMS	41 (39.05)	64 (60.95)	0 (0.00)
TET	29 (27.62)	5 (4.76)	71 (67.62)
CHL	62 (59.05)	10 (9.52)	33 (31.43)
SXT	68 (64.76)	37 (25.34)	0 (0.00)
CFZ	44 (41.90)	61 (58.10)	0 (0.00)
CTX	88 (83.81)	12 (11.43)	5 (4.76)
CAZ	93 (88.57)	12 (11.43)	0 (0.00)
CFX	96 (91.43)	9 (8.57)	0 (0.00)
GEN	89 (84.76)	6 (5.71)	10 (9.52)
IMI	105 (100.0)	0 (0.00)	0 (0.00)
NAL	67 (63.81)	0 (0.00)	38 (36.19)
AZI	103 (98.10)	2 (1.90)	0 (0.00)
Sul	42 (40.00)	33 (31.43)	30 (28.57)
CIP	58 (55.24)	34 (32.38)	13 (12.38)
AMC	64 (60.95)	41 (39.05)	0 (0.00)
CTX/C	99 (94.29)	6 (5.71)	0 (0.00)
CAZ/C	101 (96.19)	4 (3.81)	0 (0.00)
CT	71 (67.62)	11 (10.48)	23 (21.90)
PB	80 (76.19)	13 (12.38)	12 (11.43)
MIN	87 (82.86)	18 (17.14)	0 (0.00)
AN	103 (98.10)	2 (1.90)	0 (0.00)
ATM	99 (94.29)	0 (0.00)	6 (5.71)
FEP	98 (93.33)	2 (1.90)	5 (4.76)
MEM	102 (97.14)	3 (2.86)	0 (0.00)
LEV	32 (45.71)	70 (66.67)	3 (2.86)
DOX	27 (25.71)	9 (8.57)	69 (65.71)
KAN	95 (90.48)	0 (0.00)	10 (9.52)
STR	20 (19.05)	19 (18.09)	66 (62.86)
GMI	63 (60.00)	42 (40.00)	0 (0.00)

**Table 3:** Drug resistant spectrum of 105 *Salmonella* strains collected from 2018 to 2020 in six sentinel hospitals from Huzhou, Zhejiang, China.

Antibiotic resistant species (species)	Drug-resistant spectrum	Number of isolates (*n*)	Proportion (%)
3	TET-DOX-STR	5	4.76
	TET-CHL-DOX	1	0.95
	AMP-TET-DOX	1	0.95

4	TET-Sul-DOX-STR	1	0.95
	TET-CHL-Sul-STR	1	0.95
	NAL-CT-PB-STR	1	0.95
	AMP-NAL-CT-STR	2	1.90
	AMP-NAL-CT-PB	1	0.95
	AMP-TET-DOX-STR	1	0.95
	AMP-TET-CHL-DOX	4	3.81
	AMP-TET-DOX-STR	3	2.86
	TET-Sul-DOX-STR	1	0.95

5	AMP-TET-Sul-DOX-STR	8	7.62
	AMP-NAL-AZM-FEP-KAN	1	0.95
	AMP-NAL-Sul-CT-STR	2	1.90
	TET-CHL-Sul-CIP-DOX	1	0.95
	AMP-TET-NAL-DOX-STR	5	4.76
	AMP-TET-NAL-Sul-DOX	1	0.95
	AMP-TET-CHL-Sul-DOX	1	0.95
	AMP-TET-CHL-DOX-KAN	1	0.95
	AMP-NAL-CT-PB-STR	5	4.76
	AMP-TET-CHL-DOX-STR	3	2.86
	AMP-TET-NAL-Sul-DOX	1	0.95

6	AMP-TET-CHL-Sul-DOX-STR	2	1.90
	AMP-TET-CTX-NAL-Sul-DOX	1	0.95
	AMP-TET-NAL-Sul-DOX-STR	1	0.95
	AMP-TET-CHL-GEN-DOX-STR	1	0.95
	AMP-TET-CHL-GEN-CIP-DOX	1	0.95
	AMP-TET-CTX-AZM-DOX-STR	1	0.95
	AMP-TET-NAL-CT-DOX-STR	1	0.95

7	AMP-TET-CHL-Sul-AZM-FEP-DOX	1	0.95
	AMP-TET-CHL-NAL-Sul-CIP-DOX	1	0.95
	AMP-TET-NAL-CT-PB-DOX-STR	5	4.76
	AMP-TET-CHL-Sul- DOX-KAN-STR	1	0.95
	AMP-TET-CHL-GEN-Sul-DOX-STR	1	0.95
	AMP-TET-CTX-AZM-FEP-DOX-STR	1	0.95
	AMP-TET-CHL-GEN-CIP-DOX-STR	2	1.90

8	AMP-TET-CHL-NAL-Sul-CIP-DOX-STR	2	1.90
	AMP-TET-CHL-GEN-Sul- DOX-KAN-STR	1	0.95
	AMP-TET-CHL-GEN-NAL-CIP-DOX-KAN	1	0.95
	AMP-TET-CHL-NAL-CIP-DOX-KAN-STR	1	0.95

9	AMP-TET-CHL-GEN-NAL-CIP-DOX-KAN-STR	1	0.95

10	AMP-TET-CHL-NAL-Sul-CIP-LE V-DOX-KAN-STR	1	0.95

12	AMP-TET-CHL-CTX-GEN-NAL-CIP-AZM-FEP-LEV-DOX-KAN	2	1.90

## Data Availability

Data supporting the results of our study can be found in our manuscript.
